# The effect of hospital-to-home transitional care using a digital messaging application on the health outcomes of patients undergoing CABG and their family caregivers: a randomized controlled trial study protocol

**DOI:** 10.3389/fcvm.2023.1224302

**Published:** 2023-10-31

**Authors:** Maryam Maleki, Abbas Mardani, Raziyeh Iloonkashkooli, Alice Khachian, Manela Glarcher, Mojtaba Vaismoradi

**Affiliations:** ^1^ Pediatric and Neonatal Intensive Care Nursing Education Department, School of Nursing and Midwifery, Tehran University of Medical Sciences, Tehran, Iran; ^2^ Nursing and Midwifery Care Research Center, School of Nursing and Midwifery, Iran University of Medical Sciences, Tehran, Iran; ^3^ Student Research Committee, Shiraz University of Medical Sciences, Shiraz, Iran; ^4^ Institute of Nursing Science and Practice, Paracelsus Medical University, Salzburg, Austria; ^5^ Faculty of Nursing and Health Sciences, Nord University, Bodø, Norway; ^6^ Faculty of Science and Health, Charles Sturt University, Orange, NSW, Australia

**Keywords:** nurse, cardiovascular disease, digital solution, family caregiver, home care, medication management, safe care, transitional care

## Abstract

**Objectives:**

Given the increasing trend of care transition from healthcare settings to patients’ own home, patients and their family caregivers should take more responsibilities for care at own home. This study is going to investigate the effect of a transitional care program from hospital to own home using a digital messaging application on patients’ undergoing coronary artery bypass graft (CABG) surgery and their family caregivers’ health outcomes.

**Methods:**

A parallel randomized controlled trial study will be conducted in a hospital in a metropolis located in southwestern Iran. Sampling will be performed sequentially and the eligible dyad of patients and family caregivers will be randomly assigned to intervention and control groups. The intervention group will receive a transitional care program for 8 weeks using the WhatsApp on the mobile phone based on the person-centered care approach, but the control group will receive routine care for patient’s transition. Data collection will be conducted at baseline, immediately after the intervention, and two months after the intervention using demographic questionnaire, Cardiac Self-Efficacy Scale (CSES), MacNew Heart Disease Health-Related Quality of Life questionnaire (MNHD-Q), Cardiac Symptom Scale (CSS), Morisky Medication Adherence Scale, and Caregiver Burden Scale (CBS). Descriptive and inferential statistics will be used for data analysis.

**Conclusions:**

The results of this study will allow evaluating the effectiveness of an innovative transitional care program to patients’ own home using a digital messaging application. If the transitional program is shown feasible and effective it can be incorporated into existing care programs and stimulate further studies on the use of digital solutions for improving the continuity of care in own home.

## 1. Introduction

Cardiovascular disease (CVD) is one of the most common non-communicable diseases worldwide and about 17.9 million people die each year from it, and more than three-quarters of them are in low and middle-income countries (1). In Iran, CVD is rapidly increasing, so that in 2016, 43% of all deaths were due to CVD (2).

Many patients with CVD who do not respond to medications undergo a coronary artery bypass graft (CABG) surgery (3). Although heart surgery saves patients from death, it is accompanied with adverse and durable consequences on the patient’s life. Signs and symptoms experienced by patients after a CABG surgery include angina, dyspnea, fatigue, depression, sleep problems, pain at the surgical site, swelling of the legs, cardiac dysrhythmia, anxiety, and anorexia. These consequences continue even after discharge from the hospital (4–7).

Patients undergoing a CABG surgery are not only seeking to increase their life expectancy but also to improve their quality of life (QoL). The poor QoL of these patients after surgery is associated with the severity of disease, lower survival, referral and readmission to the hospital, and reduced functional activities (8). Therefore, strategies such as providing care training and managing the patient’s physical and mental problems should be used to help them return to normal daily life and maintain optimal QoL (9, 10).

Active patient participation is one of the important principles in the chronic disease care program aiming at increasing self-management and control of risk factors (11). However, these programs are often designed by healthcare professionals, and patient participation in the preparation of care plans often is ignored (12). In this regard, the World Health Organization (WHO) has recommended person-centered care (PCC) as a key element in the high-quality care among patients with chronic conditions (13). The PCC approach focuses more on the patient, his/her needs, preferences, and values than the disease (14).

One of the basic and central concepts for patients with CVD and in the PCC approach is self-efficacy (15, 16), which influences patients’ QoL and health (17). Self-efficacy is the judgment of what one thinks on one can do (18). Theoretically, self-efficacy can be modified and interventions can change it (17).

Patients undergoing CABG surgery usually need support from their spouses or family members for postoperative care and cardiac rehabilitation. Family caregivers often experience concerns about surgical problems and uncertainty about the patient’s future health status (19, 20). A recent study found that family caregivers often face challenges after the CABG surgery including the provision of social support, managing behavioral problems, taking on extra chores at home, and monitoring patients’ symptoms (21). Therefore, caregiving’s burden experienced by family caregivers of patients undergoing the CABG surgery and the provision of appropriate interventions to reduce these problems should be addressed (22).

Proper follow-up of patients undergoing the CABG surgery in the post-discharge period based on the transitional care program from hospital to own home can reduce the readmission rate and the incidence of adverse events (6). The provision of a comprehensive and well-codified follow-up and transitional care program for patients to recover after heart surgery is crucial (23). It can discover potential and actual patient problems and provide an opportunity to use a correct method for patient care management (24, 25).

Transitional care program consisting of patient education helps maintain independence in self-care and improve QoL (26). In recent years, a special attention has been made on transitional care for the safe transfer of patients between different healthcare levels and environments to maintain continuity of care. Transitional care includes transfers between the home, hospital, other care settings such as nursing homes, and consultation with various healthcare providers in outpatient settings (27, 28). Transitional care is an important part of interventions to prevent readmission of patients to healthcare centers and the provision of home-based and integrated healthcare services (29, 30). In transitional care, the actual environment and resources available in the patient home must be considered to achieve expected outcomes (31, 32). The results of previous systematic reviews showed that transitional care interventions improved self-management skills, reduced depression and readmission, and enhanced satisfaction (33, 34).

In developed countries, follow-up programs are carried out with various tools and technologies such as the telephone, which are implemented and continued for months after discharge and even lifelong care (35). Mobile technology is increasingly used in the field of health care (36). In recent decades around the world and with the development of data networks, mobile phones have become one of the most important technologies in providing health care. This technology is used for various purposes such as remote health control and self-management of health by patients with chronic conditions. Patients can use it for self-control and information exchange in various forms and in companionship with healthcare providers (37).

In Iran, the use of smart phones and the internet has increased significantly in recent years, so that almost all Iranian families benefit from these facilities (38). However, no action has been taken to benefit from the use of smart phones and popular social media platforms such as the WhatsApp on the mobile phone for the provision of transitional care from hospital to own home in cardiovascular patients. Therefore, it is necessary to take measures so that these patients can receive education, counseling, and follow-up at own home without the need for unnecessary referrals after discharge from the hospital.

Transitional care in previous studies have been carried out mostly in the form of follow-up after hospital discharge using telephone calls and sending short messages by carious healthcare providers. They have been mostly regardless of the capacities of patients and their family caregivers to perform care duties (39–45). In our study, nurses play a key role that is beyond the follow-up or discharge program. The transition care program in our study will begin before that the patient is discharged from the hospital and is transferred to own home, and will be developed based on the PCC approach with the coordination of patients and their families according to their needs and capacities. Therefore, this study is going to investigate the effect of a transitional care program from hospital to own home using the digital messaging application on self-efficacy, QoL, cardiac symptoms, and medication adherence among patients undergoing CABG surgery and caregiving burden.

## 2. Methods

### 2.1. Design

A parallel randomized controlled trial (RCT) study will be conducted on patients undergoing the CABG surgery in a hospital in a metropolis located in southwestern Iran. This protocol study was reported using Standard Protocol Items: Recommendations for Interventional Trial (SPIRIT) (46).

### 2.2. Participants and sampling

After a review of literature (41, 42, 47), eligibility criteria for the recruitment of patients include age over 18 years, CABG surgery for the first time, no other concomitant surgery during the CABG surgery, no history of respiratory diseases, no drug and alcohol addiction, no history of seizure, no psychological and cognitive problems, living with a family caregiver (spouse, children, siblings, other relatives) in own home, being literate as ability to read and write in Farsi, having a smartphone and ability to use WhatsApp, and signing the informed consent form to participate in the study.

In addition, inclusion criteria for family caregivers will be those over 18 years of age, being close family members of the patient, living with the patient, being literate as ability to read and write in Farsi, having a smartphone and ability to use WhatsApp, signing the informed consent form to participate in the study, ability to establish effective communication, experience of patient care undergoing the CABG surgery for the first time, and no history of mental health problems. Moreover, reluctance to participate, re-hospitalization, and patient death during the intervention will be exclusion criteria.

The required sample size has been separately estimated according to the study’s variables in the literature review including the QoL of CABG patients (41); cardiac symptoms (43); cardiac self-efficacy (48); medication adherence (49). The largest sample size (35 patients in each group) belongs to medication adherence using the following formula and its parameters:

$$\begin{array}{c} N=\frac{{\left(z_{1-\frac{\alpha }{2}}+z_{1-\beta }\right)}^{2}*{\left(s_{1}+s_{2}\right)}^{2}}{{\left(\mu_{1}-\mu_{2}\right)}^{2}}\\ z_{1-\frac{\alpha }{2}}=1.96\;\;\;z_{1-\beta }=1.29 \end{array}$$

μ_1_: The mean of the dependent variable in the intervention group.

S_1_: The standard deviation of the dependent variable in the intervention group.

μ_2_: The mean of the dependent variable in the control group.

S_2_: The standard deviation of the dependent variable in the control group.

α: Type I error.

β: Type II error.

Considering 10% attrition, the sample size will be 40 dyads of patients and caregivers in each group.

**Figure 1:**
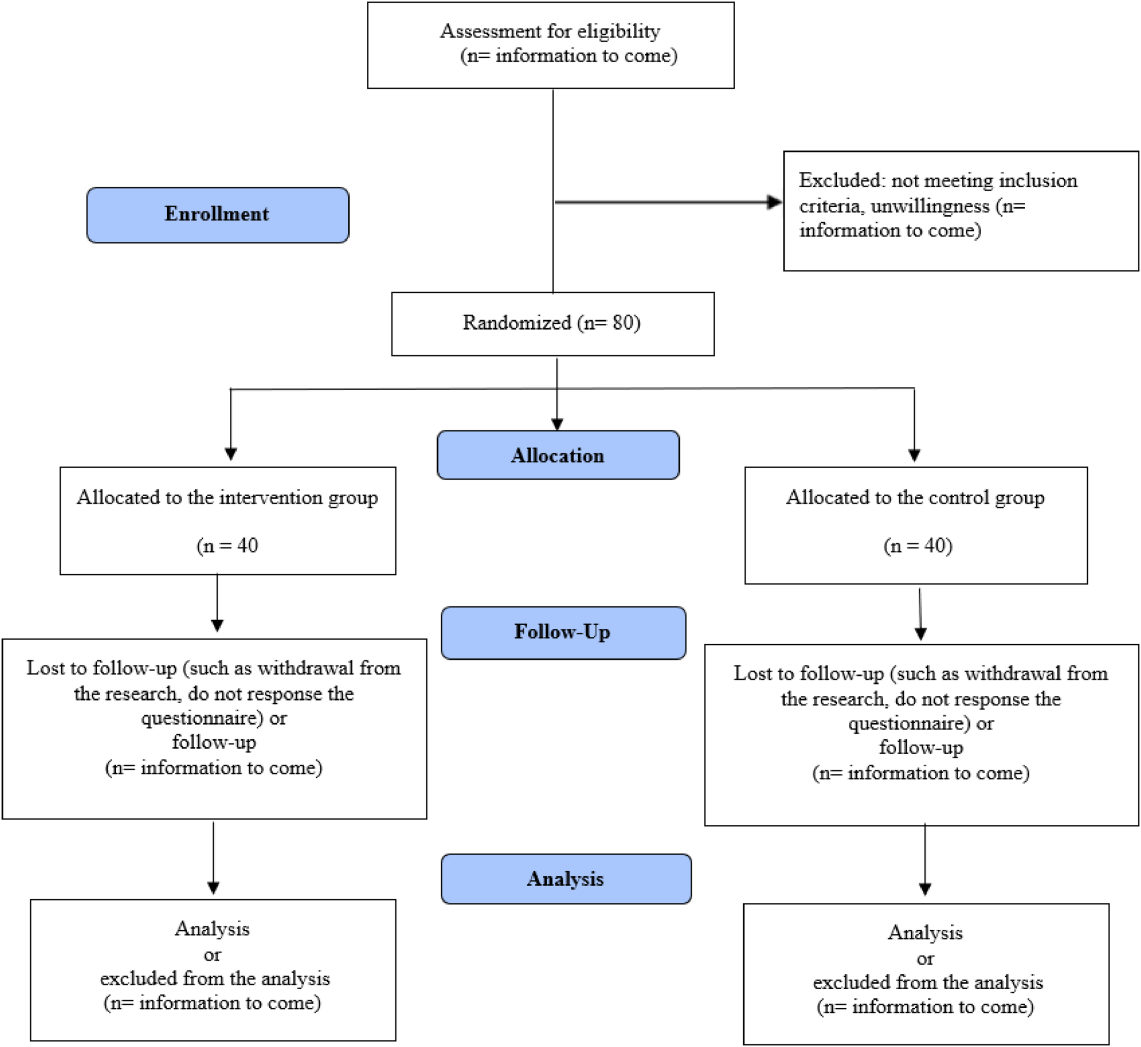
The process of the study according to the CONSORT flow diagram.

Sampling in the present study will be conducted sequentially and the dyads of the patient and family caregiver will be recruited. For the random allocation of the samples to control and intervention groups, a randomized block design of two treatments with quadruple blocks will be used. Second author (AM) will generate the random sequence using https://www.sealedenvelope.com. Sealed envelopes will also be used for concealment. An envelope will then be opened by the third author (RI) for each participant to assign them to the control or intervention group based on the card inside the envelope. The process of the study according to the Consolidated Standards of Reporting Trials (CONSORT) flow diagram has been presented in Figure 1.

### 2.3. Intervention

**Figure 2:**
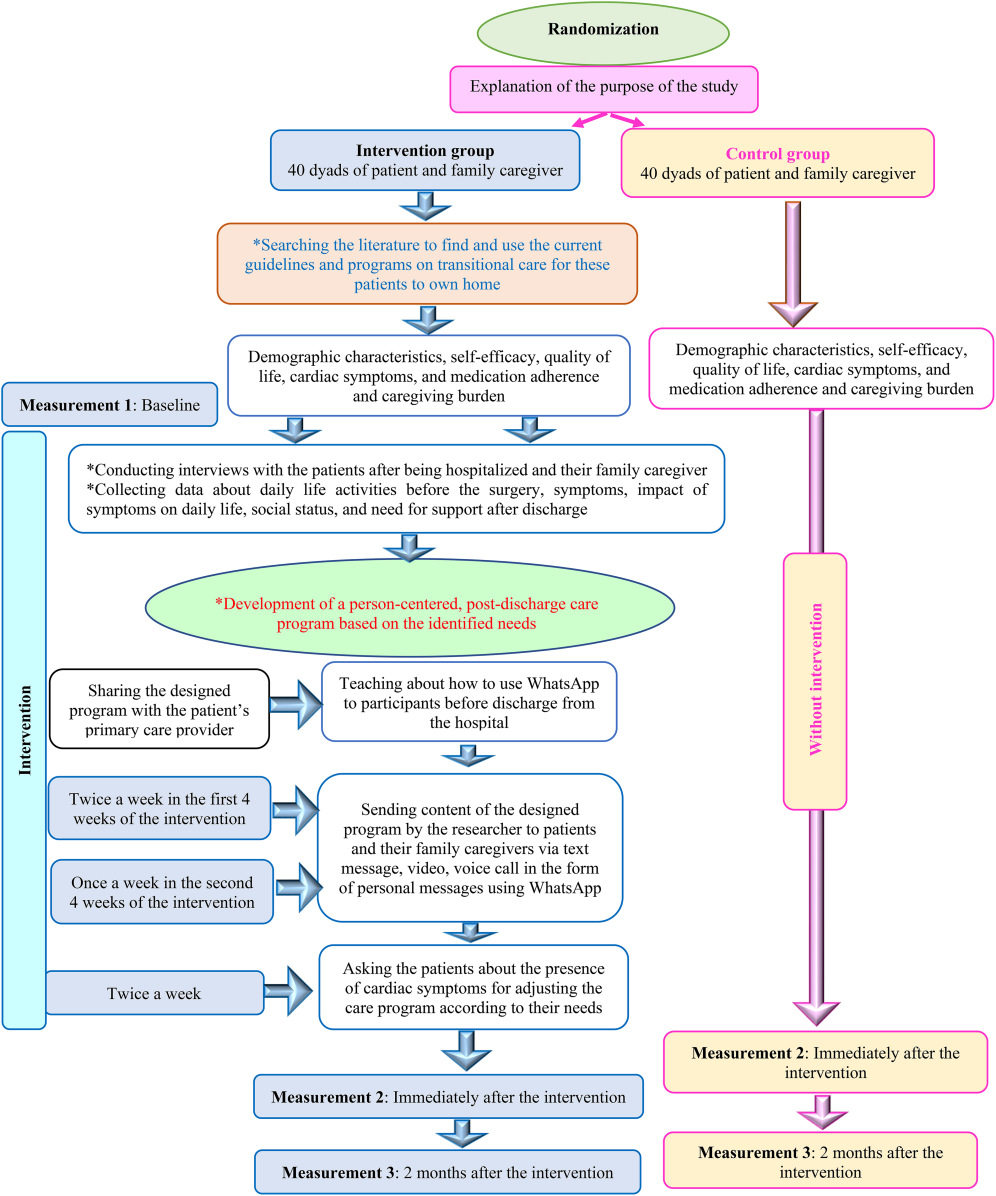
The intervention process.

Patients in the intervention group will receive an intervention designed based on the PCC approach, evidence-based practice, and clinical knowledge (Figure 2). It consists of the following aspects:

A thorough literature search will be performed to find and use current guidelines and programs on transitional care for patients undergoing cardiac surgeries to their own homes. This program should cover the areas of self-care activities, medication management, management of cardiac symptoms, dietary regimen, sexual and social activity, physical activity, wound care, control of risk factors, and referral to the primary care provider.

Interviews will be performed with patients willing to participate in the study after being hospitalized and their family caregivers to collect data about daily life activities before the surgery, symptoms, impact of symptoms on daily life, social status, and the need for support after discharge.

Development of a person-centered, post-discharge care program by the research team based on the identified needs with the cooperation of the patient and his/her family caregiver. A panel of experts consisting of the nurse, specialist physician, home care specialist, dietician, social worker, and physiotherapist will assess the program in terms of validity and comprehensiveness. Also, it will be shared with the patients’ primary care providers and their perspectives and suggestions will be sought. All participants will receive the educational content based on their needs in the mentioned areas. Before discharge from the hospital, the patients and their family caregivers will be taught about how to use the WhatsApp on their mobile phones using a two-hour education session via lecture, and active discussions.

Content of the designed program will be sent by the researcher to the patients and their family caregivers in the intervention group via text message, voice and video call in the form of personal messages using the WhatsApp. Agreements will be made with the participants regarding the sending of the program’s content via the app twice a week in the first 4 weeks of the intervention and then once a week in the second 4 weeks of the intervention. In addition, the patients twice a week will be asked about the presence of cardiac symptoms such as angina, shortness of breath, fatigue, depression, sleep problems and pain at surgery, swelling of the legs, increased heart rate, anxiety and anorexia for adjusting the care program according to their needs. Furthermore, the patients and their family caregivers will be free to share their concerns and questions with the researcher via this app, which facilities the continuity of care and follow up.

The control group will receive routine care during discharge consisting of receiving advice on cardiac symptoms’ management, physical activity, medication management, diet, and visits by the physician based on face-to-face instructions provided by the ward nurse using paper pamphlets.

Due to the nature of the intervention, it is impossible to blind the participants or healthcare providers to the assigned groups. However, attempts will be made to minimize the potential for bias by using objective outcome measures and ensuring that the data analysts will be blinded to the group assignment. The control group and the randomized allocation of participants will help with minimizing selection bias. To address compliance and adherence, we will provide participants with clear instructions on how to use the digital messaging application and encourage them to engage with the program regularly. We also will send regular reminders and prompts to encourage compliance and adherence. In addition, we will monitor the participants’ engagement and adherence throughout the study period and will provide additional support and resources to those struggling to comply with the program.

### 2.4. Data collection

The demographic characteristics questionnaire, Cardiac Self-Efficacy Scale (CSES), MacNew Heart Disease HRQL questionnaire (MNHD-Q), Cardiac Symptom Scale (CSS), and Morisky Medication Adherence Scale will be filled out by the patients. Their caregivers will also fill out the demographic characteristics questionnaire, and Caregiver Burden Scale (CBS). To protect the participants’ confidentiality, the questionnaires will be completed in person at the baseline. All questionnaires, except the demographic questionnaire, will be completed by the participants immediately after the intervention and 2 months after the intervention anonymously and confidentially based on the code assigned to each participant on the WhatsApp.

#### 2.4.1. Demographics data questionnaire

It includes questions about age, gender, income and marital status, height, weight, education level, occupation, smoking, alcohol consumption, diseases’ history, family history of heart surgery, health insurance status, and place of residence. This questionnaire will be assessed through face and content validity before the data collection.

#### 2.4.2. Cardiac self-efficacy scale (CSES)

This questionnaire consists of 13 questions in two subscales of symptom control (8 questions) and performance maintenance (5 questions) on a 5-point Likert scale. Each question is assigned a score between 0 (strongly disagree) and 4 (strongly agree). The range score of self-efficacy is 0–52. The English version of the questionnaire has been validated by Sullivan et al. A high and low score indicates a high and low level self-efficacy, respectively (50, 51). The validity and reliability of the Farsi version of CSES in patients undergoing the CABG surgery has been confirmed via a content validity index (CVI) of 91.33% and Cronbach’s alpha coefficient of 0.97 (48).

#### 2.4.3. Macnew heart disease HRQL questionnaire (MNHD-Q)

It includes 27 questions in three areas of emotional (14 questions), physical (12 questions), and social (13 questions) on a seven-point Likert scale from 1 (the worst conditions related to QoL) to 7 (the best conditions related to QoL) (52). Some questions (*n* = 12) in this questionnaire fall into more than one area. The higher score indicate a higher QoL (53). The validity of MNHD-Q has been approved in Danish, Norwegian and Swedish patients. Also, the reliability assessment of this questionnaire has been supported through Cronbach’s alpha coefficients of 0.94-0.90 (53). The internal consistency of the MNHD-Q in cardiac patients has been reported 0.73 (52). The validity and reliability of Farsi version of the MNHD-Q have been assessed and endorsed on a sample of Iranian patients undergoing the CABG surgery (54).

#### 2.4.4. Cardiac symptom scale (CSS)

It assesses ten cardiac symptoms of angina, shortness of breath, fatigue, depression, trouble sleeping, pain related to incision, swelling of legs, racing heartbeat, anxiety, and poor appetite. These symptoms are measured in three dimensions of the perception of symptoms (yes-no), evaluation of symptoms (frequency and severity), and response to symptoms (interference with physical activity and the enjoyment of life). A score of zero will be given in case of lack of symptoms for three dimensions. A score of 1 is assigned to the perception of symptoms. The scores of frequency and severity, and interference with physical activity and the enjoyment of life range from 1 to 10, and 0 to 10, respectively (43). The validity and reliability of this questionnaire have been confirmed using the CVI score of 0.9–1 and the Cronbach’s alpha coefficient of 85%–98% in patients undergoing the CABG surgery (55).

#### 2.4.5. Morisky medication adherence scale

It includes 8 items, as the first 7 questions have ‘yes’ or ‘no’ answer options. Question 8 has a 5-point Likert scale from ‘always’ to ‘never’ with scores from 0 to 4. The total score of this questionnaire varies from 0 to 8. A score less than 6 indicates low adherence, 6 to <8 is moderate, and 8 shows high adherence. The reliability of the original version of this questionnaire has been confirmed in patients with hypertension with a Cronbach’s alpha coefficient of 0.83 (56). The internal consistency of the Farsi version of this questionnaire has been confirmed with a Cronbach’s alpha coefficient of 0.69 in patients with hypertension (57).

#### 2.4.6. Caregiver burden scale (CBS)

This scale includes 22 questions with five dimensions of general burden (8 questions), isolation (3 questions), frustration (5 questions), emotional conflict (3 questions), and environment (3 questions). Each question is rated on the Likert scale of never, rarely, sometimes, and is scored from 1 to 4. A higher score indicates a greater perceived burden by the caregiver. Internal consistency of this scale has been reported 0.53–0.87 in each domain (58). The validity of the Farsi version of CBS has been approved and its internal consistency through the calculation of the Cronbach’s alpha coefficient has been reported 0.55–0.69. The stability reliability of its domains using the test and retest method has been reported 0.74–0.90 (59).

### 2.5. Ethical considerations

The study protocol has been approved by the ethics committee affiliated with Iran University of Medical Sciences (decree code: IR.IUMS.REC.1400.903). Furthermore, it has been registered on the Iranian Registration Clinical Trials (IRCT) with the code of IRCT20180113038347N2. After obtaining required permissions, the participants will be informed of the study objective and method. All measures will be taken with regard to their anonymity and data confidentiality. They can leave the study at any phase without impacting their care. The participants will be asked to sign the informed consent form before the study.

### 2.6. Data analysis

The two-sided primary statistical hypothesis will be tested as follows: transitional care program from hospital to own home using a digital messaging application has an effect on the health outcomes of patients undergoing CABG surgery and their family caregivers.

Data analysis will be conducted using descriptive and inferential statistics through SPSS statistical software version 25. Demographic and outcome data will be summarized as frequencies (percentages) or means (standard deviation) according to the level of measurement. To test the study hypotheses, the dependent variables will be cardiac self-efficacy, QoL, cardiac symptoms, medication adherence, and caregiver burden across three time points as at the baseline, immediately after the intervention, and two months after the intervention. Inferential statistical tests including repeated ANOVA test will be used. *P*-value will be set at *p* < 0.05. Furthermore, we will consider the intention to treat analysis for non-compliance or incomplete adherence (missing outcomes). Sensitivity analyses will be performed to assess the potential impact of non-response bias on our findings.

## 3. Discussion

To our knowledge, this study is the first randomized clinical trial to investigate the effect of a transitional care program from hospital to own home using the digital messaging application as the WhatsApp on the mobile phone on self-efficacy, QoL, cardiac symptoms, and medication adherence among patients undergoing the CABG surgery and caregiving burden.

Since the duration of hospitalization of patients in hospitals has decreased, many transitions to own home are unplanned and urgent. As a result, patients and their caregivers are often unprepared for what is going to happen and are mostly uncertain about their roles. However, it is expected that patients and their caregivers take a self-management role in recovering their condition along with little preparation or support (60, 61). Therefore, transitioning from hospital to own home after the CABG surgery can be challenging for patients and their family caregivers. The use of digital messaging applications can improve communication and health outcomes in various healthcare settings.

The knowledge gained from this research will contribute to develop evidence regarding whether a transitional care program from hospital to own home using the digital messaging application can provide effective support to patients undergoing the CABG surgery and their family caregivers. The outcomes of this study will inform further program development using digital solutions aiming at the improvement of QoL and wellbeing among patients with CABG and reduction of burden of care among family caregivers. In addition, future interventions and policies aiming at the improvement of transitional care for patients undergoing CABG surgery can be devised.

### 3.1. Limitations

Recruitment for this study is going to begin in April 2022 and is predicted to continue until August 2023. If any technical problem in the implementation of this research using the WhatsApp is faced, a similar internal messaging app called Eitaa will be used as replacement. In addition, possible challenges associated with the use of a digital messaging application such as disconnection and low internet speed may impact the implementation and effectiveness of the intervention. Furthermore, this study will be conducted in a single hospital due to feasibility issues, which may limit the diversity of the patient population and the generalizability of the results to other healthcare settings.

Although WhatsApp is a user-friendly App and patients and family caregivers will be taught about how to use the apps, potential factors such as technological and health literacy, user compliance, or environmental factors may affect the quality of collected data. The two-month follow-up period after the intervention may provide limited insight into the long-term effects of the transitional care program. Therefore, it is suggested that future research examine the long-term effects of the transitional care program.

## 4. Conclusion

The results of this study will allow the evaluation of the effectiveness of an innovative transitional care program based on a digital messaging application to patients’ own home. If the transitional program is shown feasible and effective, it can be incorporated into existing care programs and stimulate further studies on the use of digital solutions for improving the continuity of care in own home.
